# Lipid and smooth muscle architectural pathology in the rabbit atherosclerotic vessel wall using Q-space cardiovascular magnetic resonance

**DOI:** 10.1186/s12968-022-00897-7

**Published:** 2022-12-22

**Authors:** Erik N. Taylor, Nasi Huang, Sunni Lin, Farzad Mortazavi, Van J. Wedeen, Jamila H. Siamwala, Richard J. Gilbert, James A. Hamilton

**Affiliations:** 1 Department of Physiology & Biophysics, Boston University School of Medicine, Boston, MA USA; 2 Department of Radiology, UNM School of Medicine, The University of New Mexico, Albuquerque, NM USA; 3 Department of Biomedical Engineering, Boston University, Boston, MA USA; 4 Department of Anatomy & Neurobiology, Boston University School of Medicine, Boston, MA USA; 5 AA Martinos Center for Biomedical Imaging, Department of Radiology, Massachusetts General Hospital, Harvard Medical School, Boston, MA USA; 6 Department of Molecular Pharmacology, Physiology, and Biotechnology, Warren Alpert Medical School of Brown University, Providence, RI USA; 7 Research Service, Providence VA Medical Center and Warren Alpert Medical School of Brown University, Providence, RI USA

**Keywords:** Diffusion-weighted Q-space MRI, Atherosclerosis, Cardiovascular disease, Inflammation, Cholesterol, Thrombosis, Smooth muscle architecture, Lipids

## Abstract

**Background:**

Atherosclerosis is an arterial vessel wall disease characterized by slow, progressive lipid accumulation, smooth muscle disorganization, and inflammatory infiltration. Atherosclerosis often remains subclinical until extensive inflammatory injury promotes vulnerability of the atherosclerotic plaque to rupture with luminal thrombosis, which can cause the acute event of myocardial infarction or stroke. Current bioimaging techniques are unable to capture the pathognomonic distribution of cellular elements of the plaque and thus cannot accurately define its structural disorganization.

**Methods:**

We applied cardiovascular magnetic resonance spectroscopy (CMRS) and diffusion weighted CMR (DWI) with generalized Q-space imaging (GQI) analysis to architecturally define features of atheroma and correlated these to the microscopic distribution of vascular smooth muscle cells (SMC), immune cells, extracellular matrix (ECM) fibers, thrombus, and cholesteryl esters (CE). We compared rabbits with normal chow diet and cholesterol-fed rabbits with endothelial balloon injury, which accelerates atherosclerosis and produces advanced rupture-prone plaques, in a well-validated rabbit model of human atherosclerosis.

**Results:**

Our methods revealed new structural properties of advanced atherosclerosis incorporating SMC and lipid distributions. GQI with tractography portrayed the locations of these components across the atherosclerotic vessel wall and differentiated multi-level organization of normal, pro-inflammatory cellular phenotypes, or thrombus. Moreover, the locations of CE were differentiated from cellular constituents by their higher restrictive diffusion properties, which permitted chemical confirmation of CE by high field voxel-guided CMRS.

**Conclusions:**

GQI with tractography is a new method for atherosclerosis imaging that defines a pathological architectural signature for the atheromatous plaque composed of distributed SMC, ECM, inflammatory cells, and thrombus and lipid. This provides a detailed transmural map of normal and inflamed vessel walls in the setting of atherosclerosis that has not been previously achieved using traditional CMR techniques. Although this is an ex-vivo study, detection of micro and mesoscale level vascular destabilization as enabled by GQI with tractography could increase the accuracy of diagnosis and assessment of treatment outcomes in individuals with atherosclerosis.

## Introduction

Atherosclerosis is characterized by chronic, unresolved inflammation in the arterial wall, initiated by subendothelial deposition of lipids, primarily cholesterol esters (CE) from low-density lipoproteins, that accumulate and promote progression of the plaque to advanced atherosclerosis [1–5]. It is important to detect dangerous plaques and monitor treatments before the acute event of thrombosis, which requires detailed characterization of the vessel wall at the site of lipid deposition. Smooth muscle cells (SMC), normally present in the tunica media, contribute mechanical properties to the vessel wall through contraction and the production of extracellular matrix (ECM) proteins to maintain vascular tone, blood pressure, and blood flow [6, 7]. SMC are plaque-stabilizing, in that they contribute to the formation of a fibrous cap overlying the necrotic core. However, in the presence of accumulating lipids and unresolved inflammation, SMC transform to macrophage-like foam cells and migrate to the tunica intima [8–10], which impairs their architectural and mechanical properties and leads to plaque progression, destabilization, and vulnerability to rupture [10].

Imaging the multicellular constituents of the atherosclerotic plaque to comprehend the spatially distributed “system” of cellular and biological factors that promote plaque formation has long been considered a grand challenge. High-resolution details of plaque structure and composition can be visualized by ex vivo 3D microscopic reconstruction techniques and histology, but these methods can distort both lipid and cellular organization [11, 12].

Our goal of imaging atherosclerosis is the prediction of acute events and guidance for treatment by non-invasive cardiovascular magnetic resonance (CMR) for enhanced molecular and structural observations in vivo*.* Clinical applications of CMR are enhanced by novel contrast-agents that can enable molecular imaging of specific vessel wall components at a cellular level [13]. However, new CMR contrast agents are often unsuccessful clinically because of increased complexity, costs, and side effects. To overcome these limitations, our method employs CMR with endogenous diffusion contrast to obtain information at or near all levels of cellular and tissue organization without any contrast reagents.

We specifically utilize diffusion-weighted imaging (DWI) CMR contrast mechanism to assess soft-tissue proton diffusion time and anisotropy to determine micro-scale tissue organizational properties that result from the size and position of aligned cells, proteins, and organelles. DWI and related techniques are used extensively for brain and whole-body imaging [14]. Common applications of this contrast mechanism include tumor imaging to examine diffusion restriction (tumor cellularity) [15, 16], stroke imaging [17, 18], and anisotropy of white matter fiber bundles in the brain [17–21]. Less common applications are studies of cardiac muscle [22] and vascular structure. With long diffusion imaging scan times, free molecules move out-of-phase via Brownian motion, resulting in signal attenuation, while the restriction of molecular motion results in signal retention. Free water also tends to diffuse down the principal direction of fiber orientation (down cell bodies); it cannot diffuse in other directions and is restricted to cross cell membranes. For this reason, diffusion in many tissues is anisotropic, meaning that the diffusion properties will vary depending on the orientation of objects under observation and the orientation and magnitude of the magnetic field gradient applied [20, 21]. Moreover, DWI contrast has been shown to differentiate stationary membrane lipids from fluid plaque components, particularly liquid-crystal CE droplets dispersed in atherosclerotic plaques that contribute to plaque instability [23, 24]. DWI thereby provides unique information based on differences in the diffusion times of lipids relative to protons in freely diffusing water or cells.

Long and thin cellular structures, including neurons or muscle fibers, are anisotropic at the meso-scale (intermediate between micro-scale and macro-scale) and thus with tractography can be visualized by their complex patterns [22, 25–28] and crossing fibers [29, 30] across multiple voxels. Generalized Q-space imaging (GQI) enables the collection and analysis of detailed diffusion information from DWI contrast with considerations of contributions of both the time and orientation-dependence of diffusion (Fig. 10) [25, 31, 32]. GQI offers local quantitative evaluations via the model-free voxel-wise probability distribution function (PDF) and extends beyond 2D slice-based imaging into 3D with tractography. With GQI and tractography, it is possible to examine both local and proximal 3D tissue properties at the meso-scale, including multiple cells or voxels, but less than the whole tissue [26, 33]. In our previous GQI application to cardiology, we elucidated patterns of fiber array and multiscale architecture in mouse hearts ex vivo [22].

In the current study, we combined the application of DWI acquisition methods and GQI with tractography analysis to determine the architectural signature of atherosclerosis in vessel walls in the Constantinides rabbit, a well-established preclinical model of coronary and atherosclerotic plaques at an advanced inflammatory stage and luminal thrombosis [34]. Rabbit aortas are similar in caliber to medium-sized human coronary vessels, and our histological analysis demonstrated 6 of the 8 stages categorized by American Heart Association (AHA) in human atherosclerosis, including the deposition of CE lipids, inflammation, and plaque rupture [34]. Moreover, the rabbit model of plaque progression from initiation to rupture has been well-characterized through both in vivo and ex vivo methods [2, 34–39]. Cholesterol feeding in the rabbit produces severe inflammation that does not develop with a normal chow diet. With endothelial balloon injury, some atherosclerotic plaques progress to a vulnerable stage through high lipid uptake and progressively increasing unresolved inflammation, whereas in the rabbit aorta stable plaques also develop with lower inflammation and thicker fibrous caps, which also is observed for human coronary plaques [1, 36, 37]. As a potential therapy for human disease, our recent serial CMR characterization of rabbit plaques has demonstrated decreased inflammation by the pro-resolving mediator *resolvin* [3, 37, 40] in accord with the increasingly recognized imbalance between inflammation and inflammation resolution [4]. Resolvins are bio-active molecules derived from omega-3 fatty acids that reduce inflammation from multiple sites in the body that contribute to chronic systemic inflammation in atherosclerotic plaques [37].

**Fig. 1:**
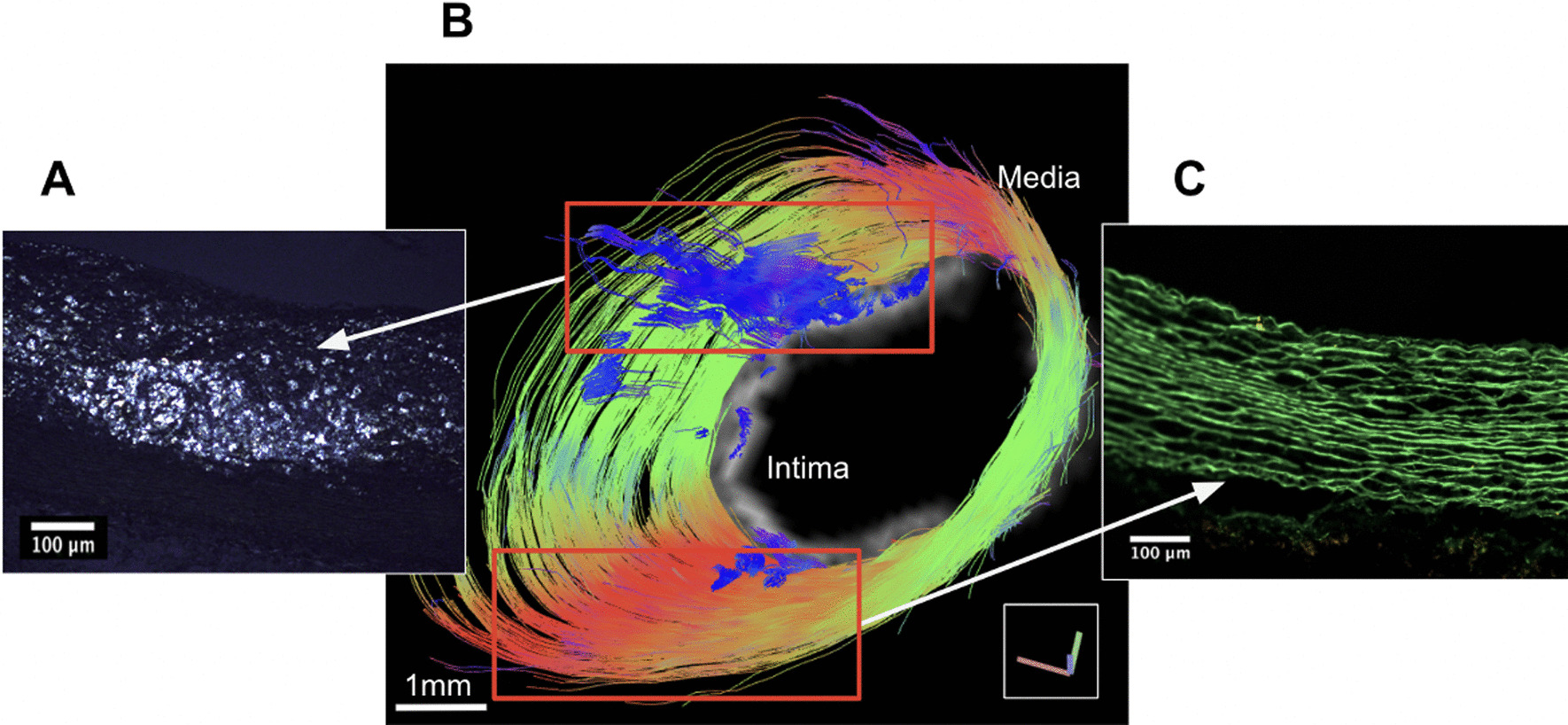
Architectural visualization of high resolution, meso-scale heterogeneity in pathologic thoracic aorta section in our rabbit model of atherosclerosis using Q-space CMR. **A** Illustrates the presence of a highly disorganized, isotropic, lipid-filled region detected by PLM. **B** Representative image of an aorta section without rupture, the green and red region of the pathologic aortic vessel wall is shown in the enlargement derived using CMR. The blue region shows a disordered region of the vessel wall containing CE, as shown by PLM in (**A**). **C** PLM of elastin tissue fibers demonstrating lipid-free, anisotropic, diffusion-restrictive organization. Color code in the CMR panel indicates the orientation of anisotropy. CE, cholesterol esters; CMR, cardiovascular magnetic resonance; *PLM*, polarized light microscopy

Here, we further assessed differences in diffusion contrast in the intimal region of abdominal aortic plaques due to the presence of CE in normal chow-fed rabbits compared to the vessel walls of cholesterol-fed rabbits with endothelial balloon injury. The presence of CE was confirmed by in situ voxel-guided cardiovascular magnetic resonance spectroscopy (CMRS) [23, 41] and was distinguished from triglycerides, which do not exhibit birefringence, by polarized light microscopy (PLM). During the same imaging session, we determined anisotropic diffusion properties of the tunica media and enlarged tunica intima by GQI. Cellular constituents and ECM properties were identified by histology and co-localized to CMR images (Fig. 1).

With GQI, we were able to visualize vessel walls with heterogeneity and observe architectural details of vessel walls not seen with 2D CMR or histology alone. The combination of relatively slow diffusion of the viscous CE in plaques with anisotropic diffusion properties in the vessel wall during progressive atherosclerosis provides an ideal environment for the application of GQI methods of analysis and thus, provides a basis for characterization in whole tissue of the complex structures that comprise the atheromatous arterial wall and the architectural features that are associated with plaque rupture.

## Methods

The data that support the findings of this study are available from the corresponding author upon request.

### Rabbit model

Groups 1 and 2 of the New Zealand White rabbits were fed a 1% cholesterol-containing chow diet for 2 months, followed by a normal diet (LabDiet, Saint Louis, Missouri, USA) for 1 month, as described previously [2, 34, 36]. Briefly, advanced highly inflamed atherosclerotic lesions were induced with the 1% cholesterol diet in conjunction with endothelial cell injury via balloon catheter procedure at 2 weeks under general anesthesia (acepromazine, 0.75 mg/kg IM; ketamine, 35 mg/kg IM; xylazine, 2.5 mg/kg IM). Group 1 (N = 5; two males and three females) were fed 1% cholesterol chow and balloon injured. Group 2 control rabbits (N = 3; female) were fed normal chow. At the end of the 3-month protocol, intraperitoneal injections (two times, separated by 48 h) of coagulation cascade factor-X activating enzyme isolated from Russell’s Viper Venom (RVV-X 0.15 mg/kg IP; Enzyme Research, South Bend, Indiana, USA) followed by histamine injection (0.02 mg/kg IV; Sigma Aldrich, St. Louis, Missouri, USA) after 30 min were used to trigger rupture of vulnerable plaques. This procedure mimics the typical rupture of human plaques. Euthanasia was carried out by acepromazine 1 mg/kg IM for 15–20 min with IV placement by ear vein, and finally administration of a lethal dose of sodium pentobarbital 120 mg/kg IV. All studies were approved by the Institutional Animal Care and Use Committees (IACUC) at Boston University School of Medicine. Both male and female rabbits were included to create the representative dataset presented herein. The sample numbers in this study are small and representative of other in vivo and ex vivo atherosclerosis studies in rabbits [2, 34, 36, 38, 39]. No differences were noted here or in our previous in vivo studies by Taylor et al. [24] or Pham et al. [36], possibly due to the surgical aspects of the study compared to sex differences during natural progression of atherosclerosis.

### High-field ex vivo 11.7T CMR and CMR

After in vivo imaging of the thoracic aorta below the lungs and including the renal branch at 3T [24] using our protocol [2], we studied “excised” segments by high field CMR. Two diffusion CMR protocols were developed and optimized to assess plaque constituents and properties. The first method was a 2D DWI acquisition with multiple diffusion-sensitizing B-values (range 0 to 4000 s/mm^2^) and an axial gradient orientation to determine the role of gradient strength and diffusion time on diffusion CMR contrast, as previously developed in our lab [23]. The 2D DWI acquisition was a standard spin-echo sequence with echo-planar imaging (EPI) read-out, TE = 22.68 ms, TR = 2000 ms, NEX = 10, 52 × 52 µm^2^ in plane resolution, 1 mm thick slices, and an intra-slice gap of 1.5 mm requiring 53 min of scan time. These experiments were first carried out in water to demonstrate the diffusion properties of free water compared to the vessel wall, with all subsequent samples being placed in the viscous CMR-signal inert fomblin liquid (Sigma Aldrich). Following initial experiments, the 3D GQI acquisition was carried out with a single B-value (selected during initial experiments) and multiple gradient orientations. The 3D GQI acquisition was used to determine the orientation dependencies of diffusion contrast with isotropic voxels. For the 3D acquisition, a 3D spin-echo sequence with a multi-shot EPI readout was used with a B-value of 2000 s/mm^2^, TE = 19.15 ms, TR = 750 ms, 91 gradient directions applied, B = 0 image acquired, and an isotropic voxel size of 150 µm^3^ (size 150 × 150 × 150 μm^3^) requiring 15 h, 20 min of scan time. Additional details about sample preparation are provided in the *Histologic validation* methods. The CMR acquisitions were followed by voxel guided CMRS of plaque constituents for identification of fluid lipids by chemical shifts. Voxel-guided PRESS was used with TE = 20 ms, TR = 2500 ms, a matrix size of 2048, NEX = 512, and a scan time of 21 min. We varied the CMRS voxel size to fit plaque or normal vessel wall regions of interest (ROI), with an average size of 5.6 ± 1.7 mm^3^. Temperature was maintained at room temperature (25 °C) within the magnet using the temperature controller, after samples were equilibrated at room temperature prior to imaging. All CMR and CMRS procedures were performed on a 500 MHz 11.7T vertical bore CMR system (Bruker, Co., Billerica, Massachusetts, USA).

### Histologic validation and correlation with images

Histology was carried out on excised rabbit aortas fixed in 4% paraformaldehyde for 24 h. After fixation, the aorta was washed in phosphate-buffered saline for a minimum of 24 h, followed by CMR. The aorta was co-registered with CMR images using the renal and other smaller branches as fiducial markers and sectioned into 10 mm segments for histology [2, 34, 36]. Direct embedding in paraffin was used followed by sectioning for Trichrome staining or for immuno-histochemistry. Since this method can remove lipids during processing, for lipid assessment, we used cryopreservation of the aorta by successive sucrose bathing (10% and 20% by weight in phosphate-buffered saline), followed by freezing and cutting into 12 µm thick cryo-sections. The frozen sections were directly stained by Oil Red O (ORO) for neutral triglycerides and CE or examined with PLM in unstained sections to identify CE droplets by their characteristic birefringence [34]. PLM was co-registered with multiple slices of CMR that do not require solvent extractions or freezing. The CMR images cover the entire volume of a particular aortic section. Co-registration was performed through a combination of scanning multiple small aorta sections in CMR, subsequently slicing these sections for various histology, and finally identifying anatomical markers that correspond to both CMR and histology in small sections. Auto-fluorescence (excitation/emission 488/510 nm) was used to detect elastin fibers. It is important to recognize as well that conventional immunohistochemistry is inherently two dimensional, whereas our images represent 3D volumes. While the reconstruction of a large number of 2D slices into an equivalent volume dataset, and its precise co-registration, is technically feasible, this generally requires considerable user interface and is therefore open to bias. We believe that our approach, combined with proper reference to the literature, is a more valid experimental approach.

### Q-space CMR and image processing

GQI is a mathematical reduction that combines the Fourier transform and the calculation of the PDF from the spin density function, a unified reference comparing the voxel-wise coordinate and diffusion displacement to enable quantitative comparisons. Image reconstruction was conducted using the DSI Studio (http://dsi-studio.labsolver.org; Pittsburgh, Pennsylvania, USA) software with GQI methods from the 3D acquisition data. The presence of enlarged plaques, which are often outwardly remodeled as seen by in vivo CMR in our rabbit model [2, 34, 36], was identified by a combination of CMR and processed images from GQI. ROIs were drawn on GQI images in DSI Studio and labeled as either ‘intima’ or ‘media’ in five consecutive slices. The PDF method of GQI enables quantification of voxel-based statistics from the ROI, including generalized fractional anisotropy (GFA), isotropic diffusion component (ISO), restricted diffusion index (RDI), and non-restricted diffusion index (nRDI). Diffusion tensor imaging (DTI) fractional anisotropy (DTI-FA) was also calculated for comparison to GQI methods. GFA is a diffusion anisotropy index calculated per voxel from the orientation distribution function (ODF) and is recommended instead of DTI-FA for quantification of anisotropy at higher b-values and in regions of heterogeneous fiber configuration [42]. The ISO value is the calculated minimum distribution value of the ODF [31]. The RDI index quantified the density of restricted diffusion and the nRDI index quantified non-restricted diffusion given a diffusion sampling length ratio of 1.25 and 0.25, respectively [43]. The GQI method also determines the dominant orientation of anisotropy in the imaging space of each voxel and the presence of crossing or overlapping fibers within the voxel. In our evaluations, when coherence was consistent across multiple voxels, tracts were drawn. For tractography generation, 50,000 tracts were generated with an angular threshold of 35° and with Euler streamlining used to determine fiber trajectories across multiple voxels [25–27]. Tractography results are shown as color coded for fiber orientation, unless otherwise noted in the figure legend.

### Statistical analysis

Individual voxel-based statistics were calculated from ROI drawn in DSI Studio. Statistical comparison was then carried out using the R programming language and RStudio (https://rstudio.com/; Boston, Massachusetts, USA). Statistical analysis was performed using the Shapiro–Wilk test for normality, followed by the f-test for variance, and finally the one-sample t-test comparing intima to media. A p-value less than 0.05 was considered significant under each statistical test. Graphics were output as individual readings from animals.

## Results

### Image reconstruction of vessel wall and atherosclerotic plaque from 3D data acquisition with GQI quantification and color coding for fiber orientation

**Fig. 2:**
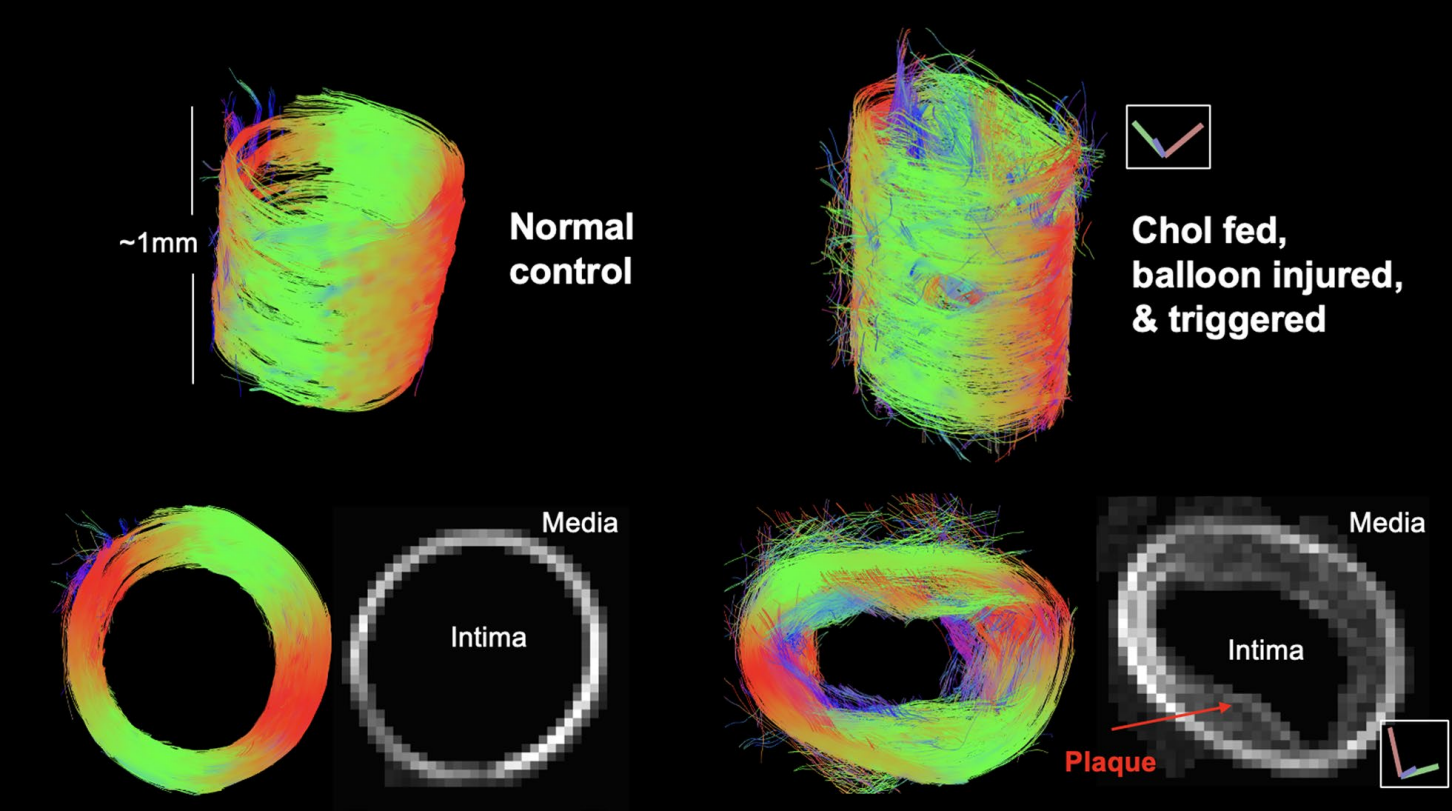
Representative image reconstruction of abdominal aorta. Color coded 3D acquisition data with GQI quantification and 2D CMR slices reveal a thicker vessel wall in the atherosclerotic diseased rabbit. Two representative segments cut from the normal and atherosclerotic aorta are shown. The normal vessel wall shows a well-defined vessel wall orientation with no irregular fiber details. The cholesterol (CHOL)-fed vessel wall shows an advanced plaque without rupture in this segment. The 3D longitudinal oblique views on top; axial view of 3D in bottom left; 2D CMR slice on bottom right. The 3D image does not provide an accurate measure of the vessel wall thickness because the wall is not perfectly straight, and the thickness is variable along the lengths of the vessel. The 2D image gives an accurate thickness in a thin axial slice. The color in the figure shows the XYZ fiber orientation of GQI. *GQI*, generalized Q-space imaging;

Tractography analysis by GQI yielded remarkably different and valuable images from 2D CMR. Figure 2 shows the 3D GQI tractography results with longitudinal and axial views of a chow-fed rabbit without atherosclerosis and a cholesterol-fed, balloon-injured rabbit with advanced atherosclerosis. In general, tractography of the aorta showed highly organized, circumferential fibers with green and red in both groups. The cholesterol rabbit showed heterogeneous atherosclerotic plaque in the intima and media and disorganization of the vessel wall fiber structure. The normal vessel wall showed a thin, homogeneous vessel wall in a 2D CMR slice. In addition, low signal area in some CMR shown is triglyceride outside of the vessel. Even though fat suppression was used, triglycerides did not provide much signal. Additionally, some of the GQI images are 3D models in perspective view, so that would account for some differences in the images. Supplemental histology for these two animals is shown in Fig. 11.

### Ex vivo 2D CMR images of the normal and atherosclerotic rabbit aorta with histological correlation

**Fig. 3:**
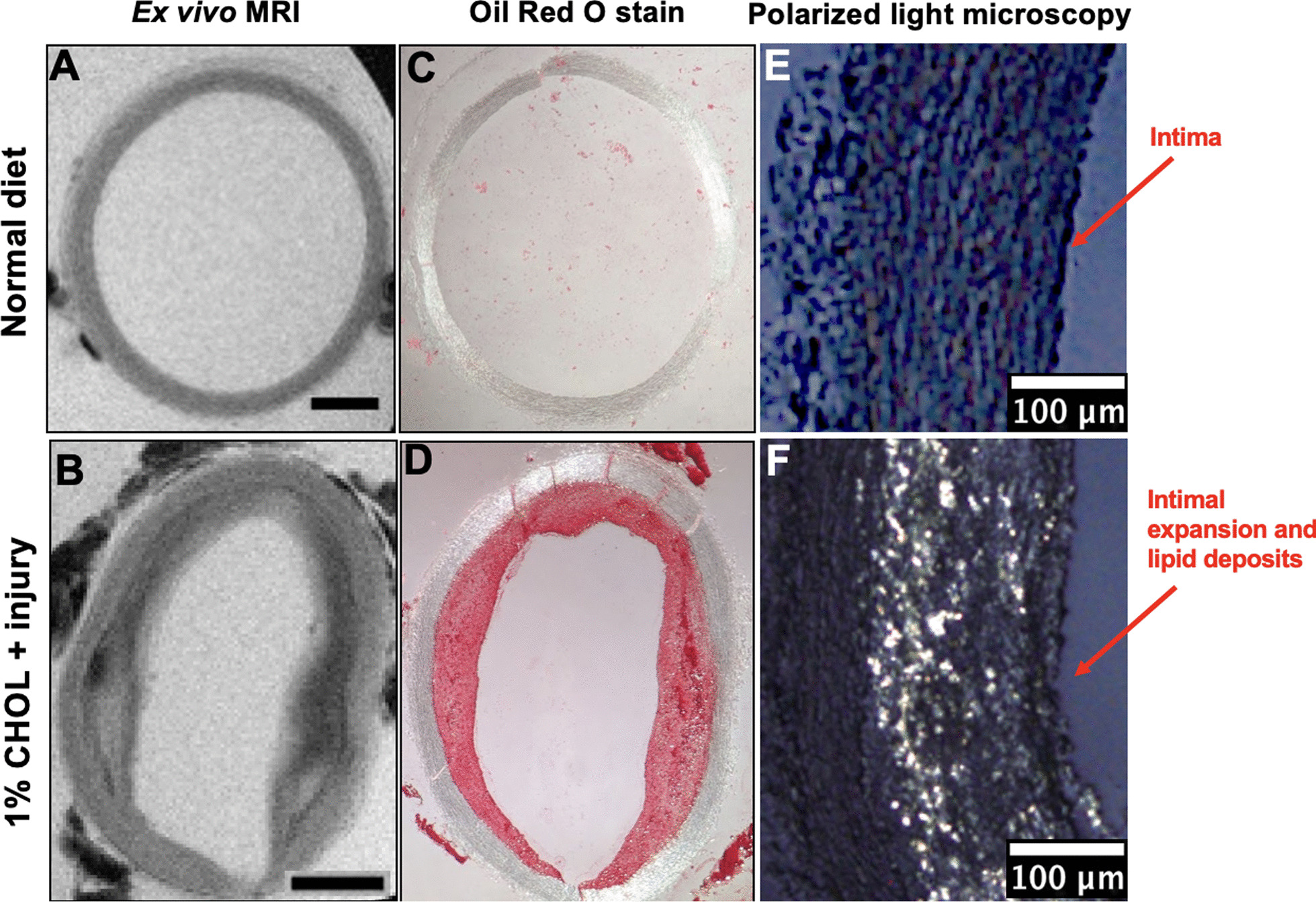
Axial ex vivo 2D CMR slices with histological validation. Top Row (**A**, **C**, and **E**) rabbits fed a normal diet. Bottom row (**B**, **D**, and **F**) rabbits fed 1% cholesterol diet + injury. CE liquid–crystal droplets are visualized by PLM in **E** and **F**. Intimal expansion and lipids were found in the 1% cholesterol diet + injury rabbit aorta compared to rabbits fed normal chow diet. In **A** and **B**, scale bars measure 1 mm

The healthy vessel walls from rabbits fed a normal chow diet are illustrated in the top row of Fig. 3. These walls are thin, and the intima and media are not well differentiated by either CMR (Fig. 3A, B) or histology (Fig. 3E). In rabbits fed 1% cholesterol diet + injured (Fig. 3B, D, F), the vessel walls are heterogeneous and thickened [2, 34, 36]. Ex vivo CMR (Fig. 3B) shows heterogeneous signal intensities that reflect different compositions and structures in the vessel wall. Histology of frozen sections with ORO staining for all lipids (Fig. 3D) corresponds to the regions that showed abundant CE by histology with PLM in those fed 1% cholesterol diet + injured (Fig. 3F).

### Cellular organization within the atheromatous vessel wall as determined by GQI with corresponding ORO staining and histology

**Fig. 4:**
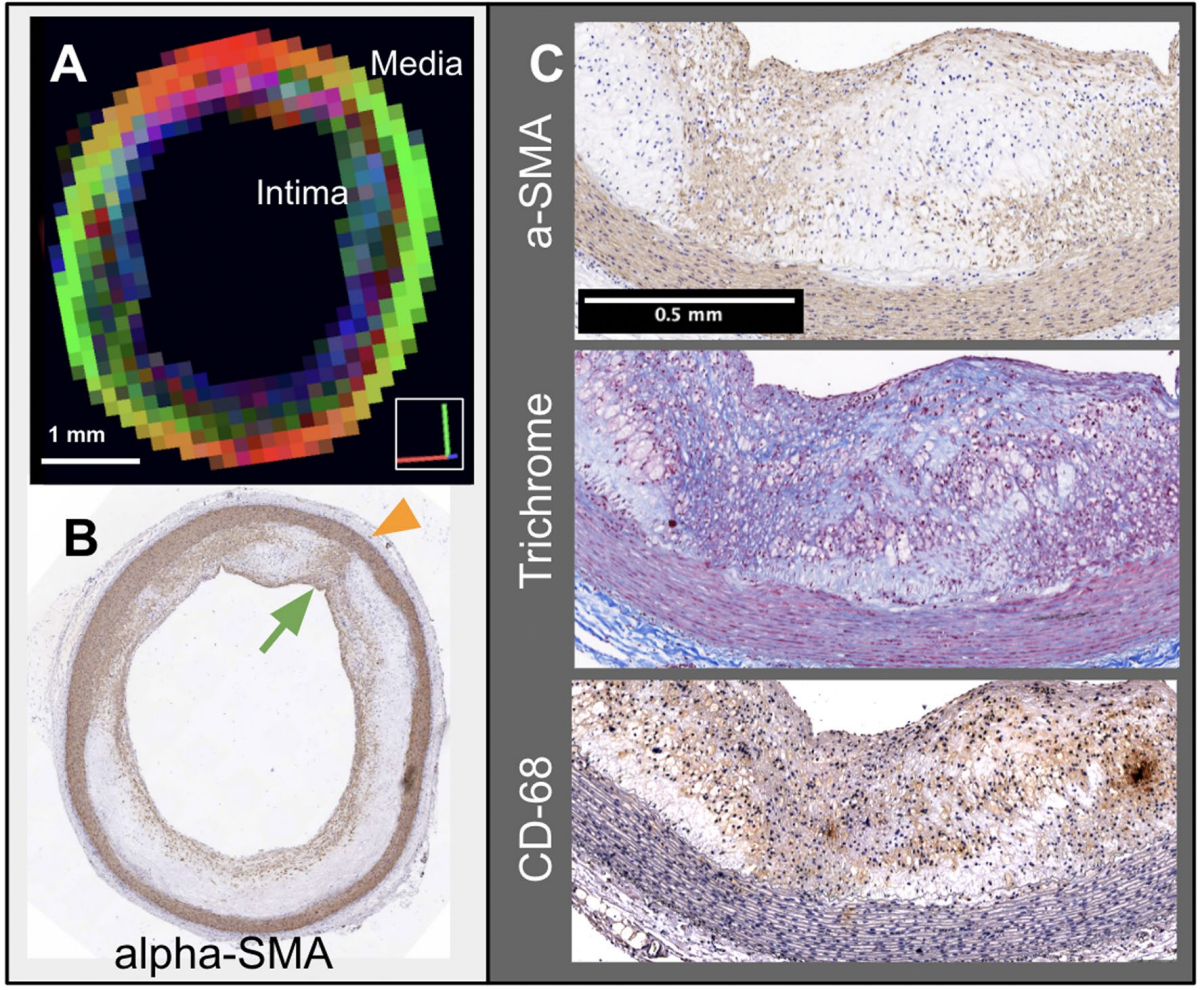
Comparison of GQI (**A**) to histology with smooth muscle cell (SMC), extracellular matrix (ECM), and inflammatory cellular components in (**B**) and (**C**) from the same abdominal aorta section in 1% cholesterol diet + injury rabbit with thickened intima. The diffusion orientation in the vessel wall is related to the orientation of SMC. **A** The color in the 2D GQI slice contrasts fiber orientation between the intima and media via diffusion signal orientations, as in Figs. 1 and 2. **B** ɑ-SMA staining of a section from the same location. **C** Immunostaining of the sections with ɑ-SMA, trichrome, and CD-68. These sections show high heterogeneity and inflammation at a molecular level

We applied GQI to visualize vessel wall regions with details of fiber orientations as shown in Fig. 4. Fiber orientation was not observable by 2D CMR or histology alone (as in Fig. 3). Using GQI, Fig. 4A shows a 2D axial section of a heterogeneous vessel wall from a 1% cholesterol diet + injury rabbit. The color-coded different diffusion orientations reflect heterogeneity of the orientation of SMC in the vessel wall in the SMC-derived foam cells (intima) and lipid-poor SMC (media) (voxel size = 150 µm^3^), but not specific chemical constituents. We used histology to achieve higher resolution and to show the corresponding cellular and molecular components that give rise to the heterogeneity observed in GQI. Figure 4B shows the matching histology slice to Fig. 4A. The arrowheads in Fig. 4B indicate the region selected for consecutive histology slices in Fig. 4C: ɑ-SMA immuno-staining shows SMC (brown) and nuclear contents (blue); trichrome staining shows (foamy pink cellular infiltrates) and collagen (blue); and CD-68 immuno-staining shows macrophages and inflammation (brown). In composite, heterogeneous regions of SMC infiltrate were found in the enlarged intima along with high inflammation.

**Fig. 5:**
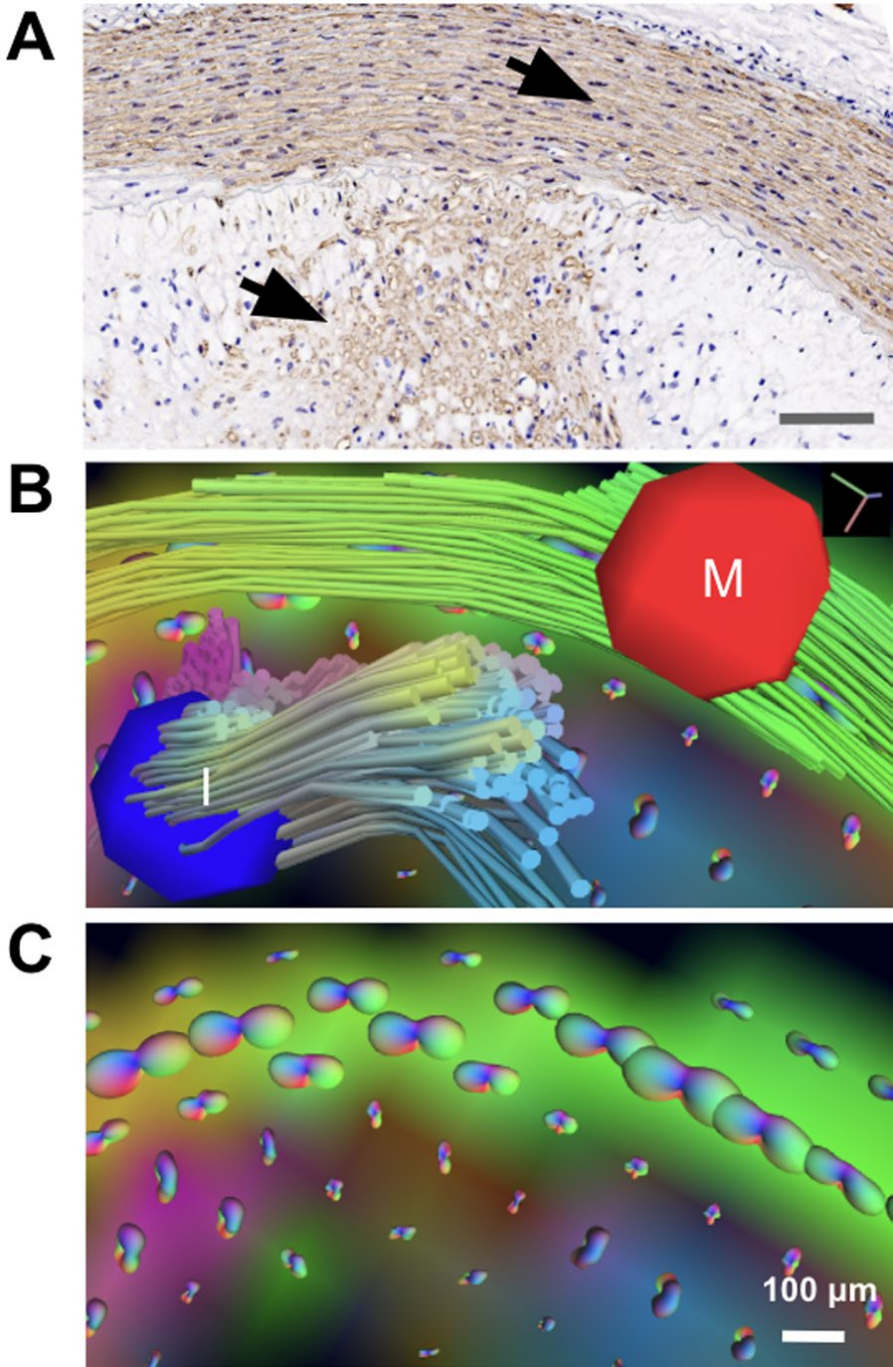
Visualization of smooth muscle cells (SMC) in the same 1% cholesterol diet + injury rabbit from Fig. 4. **A** ɑ-SMA histology, **B** GQI with tractography showing circumferentially aligned SMC in the media (M) with regions of heterogeneity in the intima (I), and **C** reconstructed voxel scale diffusion PDF

Figure 5 illustrates the different cellular orientations in the intima and the media regions in the 1% cholesterol diet + injury rabbit imaged in Fig. 4. SMC in the intima and media are seen as pink regions with ɑ-SMA histology in Fig. 5A. Our diffusion imaging methods showed circumferentially aligned SMC in the media (Fig. 5B, green) that were parallel with elastin and collagen fibers and organized perpendicular to the direction of blood flow. The diffusion signal orientation was varied, indicating regions of heterogeneity, SMC migration, and cellular disorder, as shown in detail above in Fig. 4. GQI (Fig. 5B) demonstrated cellular orientation and meso-scale architecture across the imaging regions. Two ROIs (Fig. 5B solid red, solid blue) were drawn for tractography generation (Fig. 5B) and PDFs (Fig. 5C) to contrast fiber orientations from the intima (I) and media (M). Oriented meso-scale regions were found in both the medial layer of the vessel wall and the enlarged intima, with displayed micro-scale anisotropic structure and multi-millimeter range coherence shown as tracts colorized to 3D orientation. The reconstructed voxel scale diffusion PDF is shown in Fig. 5C. Circumferential alignment of SMC in the media (green region) was represented by a coherent dumbbell-shaped PDF. In contrast, the atherosclerotic intima (multicolored region below the media) consisted of a mixture of longitudinally aligned or incoherent PDF shapes. Further details of the same vessel wall are elegantly visualized in a long axis view by diffusion tractography as shown in Fig. 12.

### GQI measures of anisotropy

**Fig. 6:**
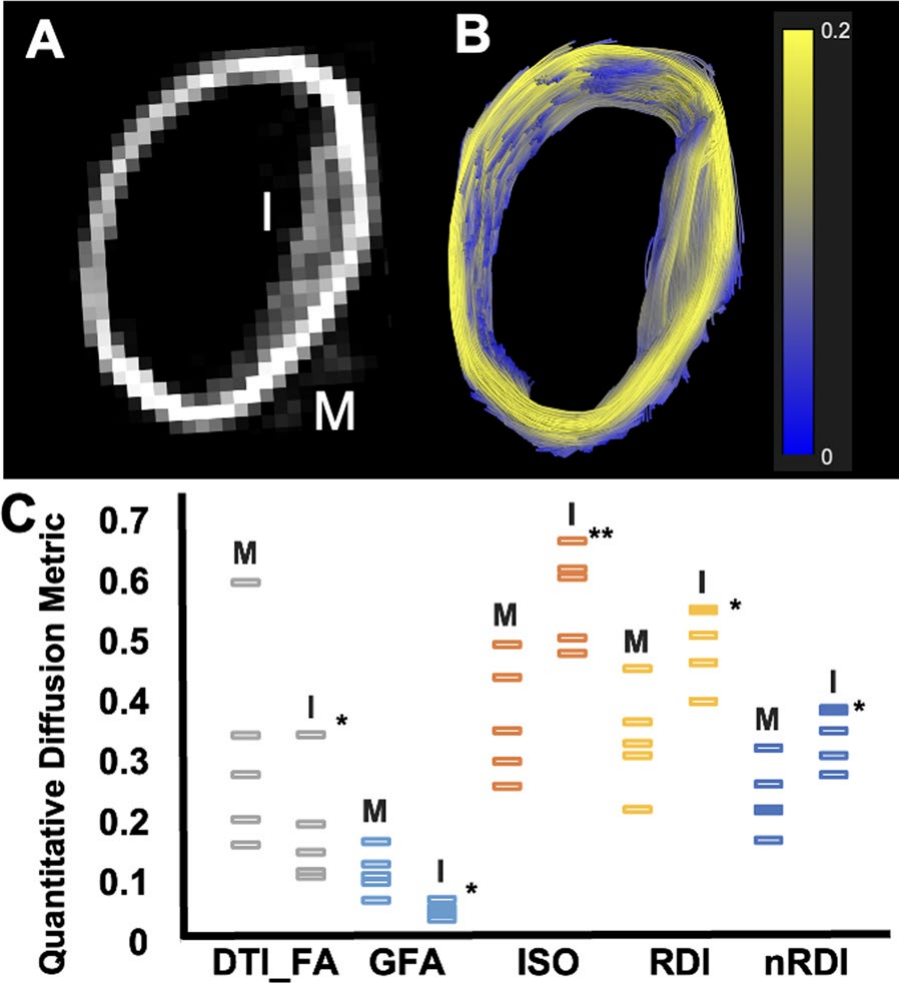
2D CMR in (**A**) and (**B**) and quantification of generalized Q-space imaging (GQI) voxel-wise metrics in the 1% cholesterol diet + injury rabbit aorta (N = 5). **B** Shows representative 3D tractography image color coded to GFA (scale bar shown) generated from ROIs of the vessel wall layer. While the enlarged intima (“I”) in (**A**) had lower diffusion anisotropic properties than the media (“M”), quantitative analysis by GQI metrics (**C**) demonstrated higher RDI, ISO, and nRDI of the intima. **B** The 3D rendered ROI tractography CMR of the same vessel wall section in (**A**) with colors differentiated by diffusion anisotropic properties. DTI-FA shows equivalent trends of higher anisotropy in the media, but with higher standard deviation between readings compared to GFA. Colors in this figure are representative of GFA as quantified here. Statistical analysis performed using the paired t-test is shown with the p-value indicated as * less than 0.05 and ** less than 0.01. *DTI-FA*, diffusion tensor imaging fractional anisotropy; GFA, generalized fractional anisotropy; *ISO*, isotropic diffusion component; *nRDI*, non-restricted diffusion index; *RDI*, restricted diffusion index

Details of anisotropic and diffusion restrictive properties were further evaluated with quantification of GQI metrics. These results are shown with individual readings from GQI metrics derived from ROIs in the media and intima from Fig. 6. Metrics which are defined in the “Methods” section are quantified and defined in Table 1 (Appendix).

**Table 1:** Metrics provided by GQI analysis of the 1% cholesterol diet + injury rabbit aorta comparing the media and intima

GQI measure	Media; intima (mean ± standard error)	Shapiro–Wilk normality test (P-value)	F-test for variance (P-value)	Two sample t-test (P-value)
DTI_FA	0.317 ± 0.077; 0.184 ± 0.043	0.051	0.288	0.168
GFA	0.116 ± 0.016; 0.054 ± 0.005	0.274	0.057	0.007
ISO	0.370 ± 0.043; 0.576 ± 0.035	0.690	0.705	0.006
RDI	0.337 ± 0.038; 0.496 ± 0.029	0.724	0.613	0.010
nRDI	0.240 ± 0.026; 0.343 ± 0.021	0.757	0.726	0.015

GQI metrics were abbreviated as generalized fractional anisotropy (GFA), isotropic diffusion component (ISO), restricted diffusion index (RDI), and non-restricted diffusion index (nRDI). The diffusion tensor imaging fractional anisotropy (DTI-FA) was also calculated for comparison to GQI methods. Statistical analysis was performed using the Shapiro–Wilk test for normality, followed by the F-test for variance, and finally the Two-sample t-test. A P value less then 0.05 was considered significant under each statistical test

#### Quantification of tract-based helix angles in 1% cholesterol diet + injury rabbits

**Fig. 7:**
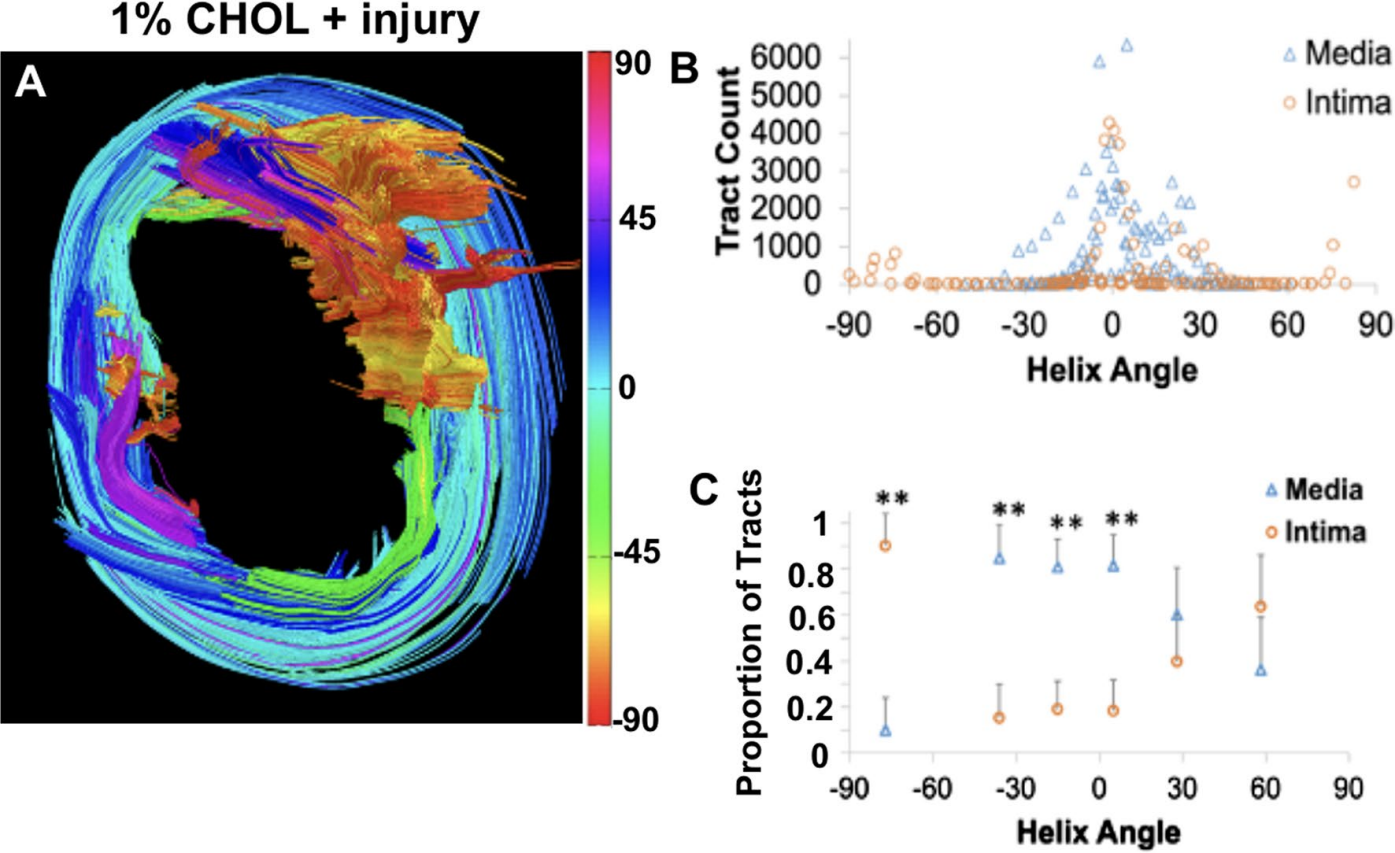
Generalized Q-space imaging (GQI) tractography of an aorta segment advanced atherosclerotic plaque without rupture using tract-based helix angles. **A** GQI displaying heterogeneity in plaque region, **B** distribution of tract count in the media and intima with regards to fiber helical angle orientation, **C** statistical evaluations of proportion of tracts with helical angle orientations. Fiber orientations via helical tractography are differentiated by the color bar as shown. Statistical analysis performed using the paired t-test is shown with the p-value indicated as ** less than 0.01

The remarkable heterogeneity of an advanced atherosclerotic plaque is illustrated by color coding GQI tractography to the helix angle (Fig. 7), with the assumption that fibers in arterial structures are oriented in a helical trajectory. A representative image of an aortic segment from the 1% cholesterol diet + injury rabbit (Fig. 7A) revealed long, organized circumferential tracts (blue and green) and highly disorganized regions (red and yellow). Quantification of fiber orientation by helix angle demonstrated a higher tract count (Fig. 7B) and a larger proportion of tracts (Fig. 7C) deviating from circumferential (helix angle = 0°) towards longitudinal (helix angle = ± 90°) orientation in the enlarged intima compared to the media.

#### Using GQI to characterize vessel wall and thrombus formation after plaque rupture

**Fig. 8:**
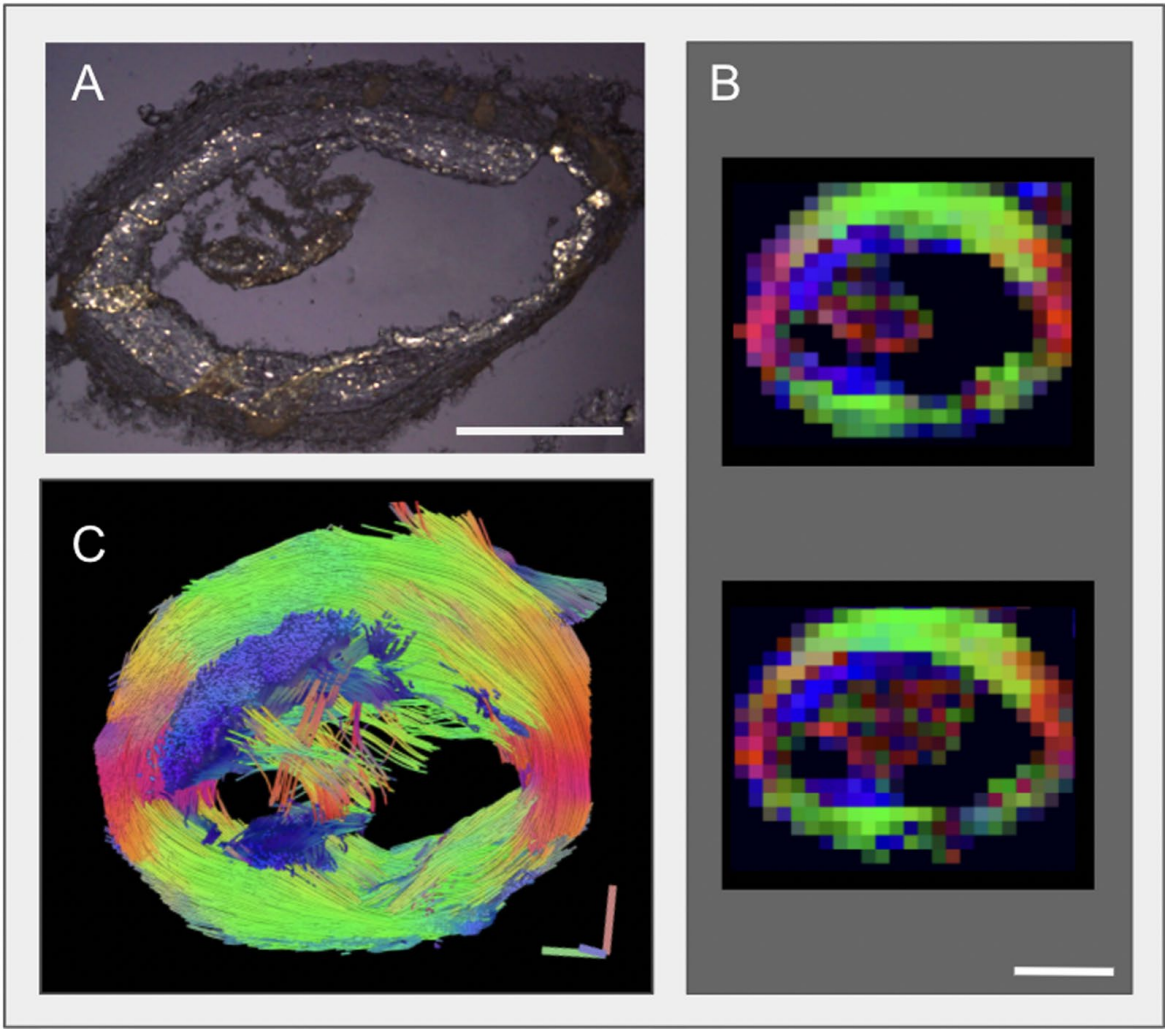
Visualization of thrombus formation in an aorta segment with plaque rupture using generalized Q-space imaging (GQI) in the 1% cholesterol diet + injury rabbit. Thrombus visualized under PLM (**A**) demonstrated the presence of lipids in both the enlarged intima (plaque) and in the thrombus. **B** 2D GQI slices of the thrombus with individual voxels shown, color encoded to fiber orientation along the given orientation axis. The thrombus propagated along the vessel wall while remaining attached to the plaque areas (blue). **C** 3D tractography reconstruction of the vessel wall and thrombus with the measured fiber orientations (axial/top view of the 3D model). The vessel wall media (green and red) and plaque regions demonstrated long-range coherence of fiber orientation, whereas the thrombus was incoherent, with short tracts of random orientation. The scale bars size in (**A**) and (**B**) is 1 mm

The *Constantinides* rabbit is a unique model for plaque rupture that resembles human atherothrombosis [35]. Regions of thrombus formation were visualized in vivo and ex vivo in the 1% cholesterol diet + injury rabbits after vulnerable plaque rupture by pharmacologic triggering (Fig. 8). The thrombus protruded into the lumen that was attached to the thick intimal wall (Fig. 8A). Substantial CE deposits (shown by histology with PLM in Fig. 8A) were present in both the enlarged intima (plaque) and in the thrombus as we observed previously [34]. In Fig. 8B, CMR slices of the thrombus are color encoded to 3D fiber orientation, as in previous figures. Contiguous GQI images showed that the thrombus adhered to a region of intimal expansion with diffusion anisotropic orientation parallel to blood flow (Fig. 8B). The thrombus propagated along the vessel wall, while remaining attached to the plaque areas (blue). Figure 8C shows a reconstructed view of the vessel wall and thrombus with tractography of the measured fiber orientations (axial/top view of the 3D model). Both the vessel wall media (green and red) and plaque regions demonstrated long-range coherence of fiber orientation, as long coherent tracts, whereas the thrombus was incoherent, as displayed by random anisotropic orientation (Fig. 8B) and short tracts in tractography (Fig. 8C). Quantification of GQI (Table 1) was carried out to determine the degree of anisotropy and diffusion restrictive properties with respect to depth in the atherosclerotic vessel wall.

### Identification of lipid constituents in DWI with CMRS

**Fig. 9:**
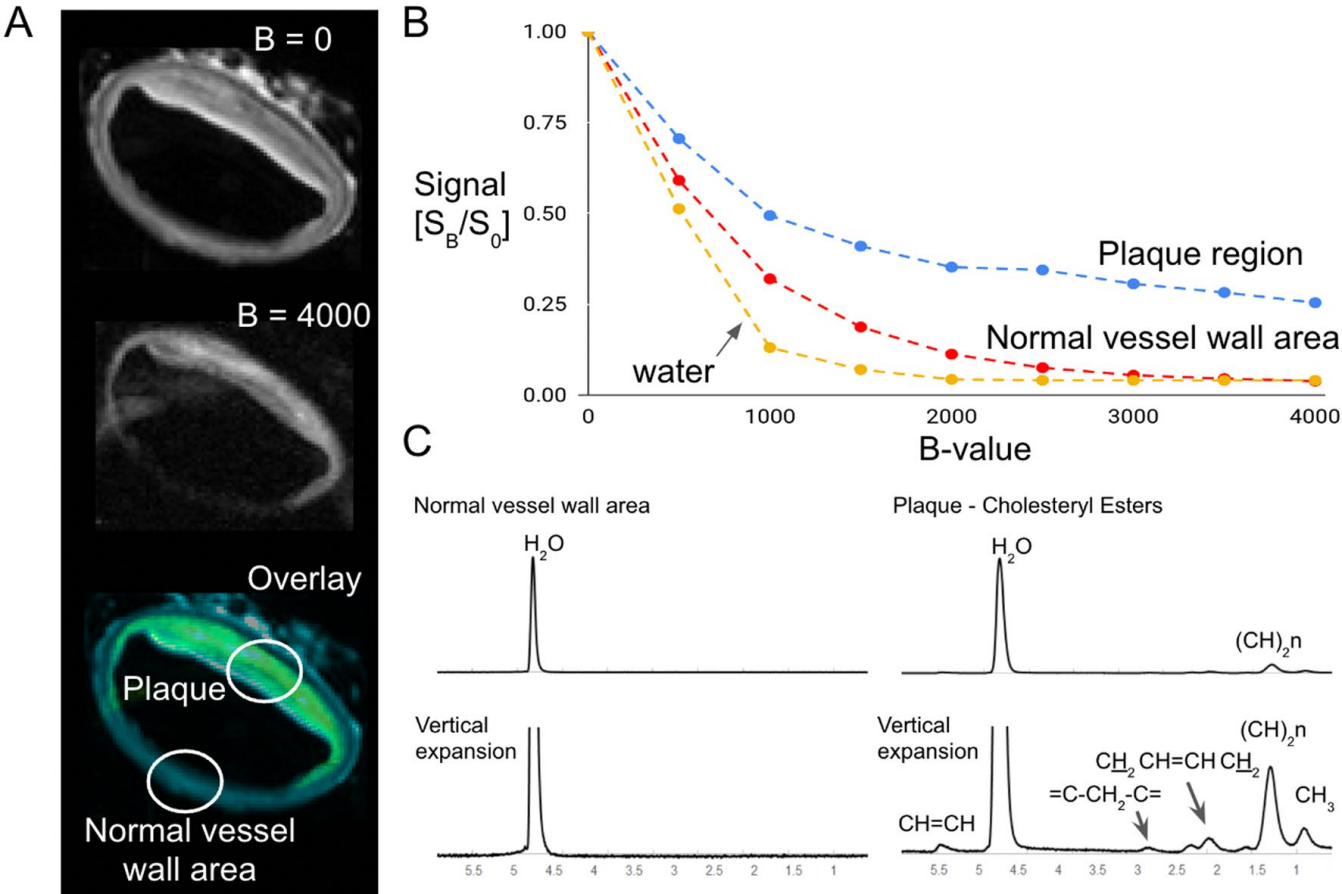
Plaque components in diffusion weighted imaging (DWI) with confirmation of plaque constituents by voxel guided CMRS in the pathologic 1% cholesterol diet + injury rabbit. **A** Lipids in the vessel wall remain hyperintense at a high B-value, while normal vessel wall constituents become attenuated. The overlay image compares B = 0 (no diffusion weighting, displayed in blue) with a semi-transparent overlay of B = 4000 s/mm^2^ (displayed in yellow). The black background is due to placing the aorta in CMR signal-inert fomblin liquid. **B** Comparison of confirmed plaque regions, normal vessel wall, and free water by normalized signal loss at increasing diffusion-weighted B-values. DWI was applied to vessel wall regions with multiple B-values (increments of 500 s/mm^2^), where increasing B-values are proportional to increased diffusion times. **C** The presence of CE was confirmed by CMRS in diseased and normal aorta regions. Lipid peaks were not detected in the normal vessel wall area (only water), while multiple lipid peaks were observed in the plaque region with a resonance profile confirming CE

**Fig. 10:**
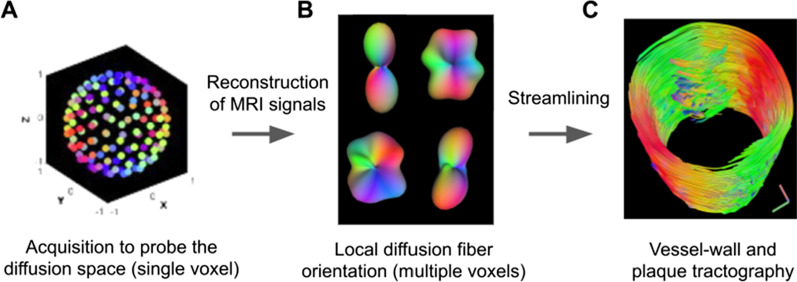
A schematic of GQI acquisition, processing, and results used here to distinguish and represent 3D fiber architecture. This method for vessel wall imaging relies on unique acquisition and image processing techniques to produce high angular resolution 3D images of intra-voxel fiber geometry. The colors represent differing angles of the fibers. **A** During acquisition, CMR is used to probe the diffusion space (Q-space) with multiple applied magnetic field gradient orientations, as represented by points on the unit sphere. **B** GQI, an acquisition and processing pipeline, enables the analysis of voxel-wise 3D diffusion anisotropy. The output results of GQI analyses are the PDFs of local diffusion. Representative PDFs are shown. **C** The anisotropic diffusion properties are linked via multiple PDF maxima and displayed as tracts, indicative of the meso-scale fiber architecture present in the vessel wall, with cellular and ECM fiber component diffusion orientation assessed with this method. Tracts are colorized with encoding according to the orientation in 3D space (inset axis)

**Fig. 11:**
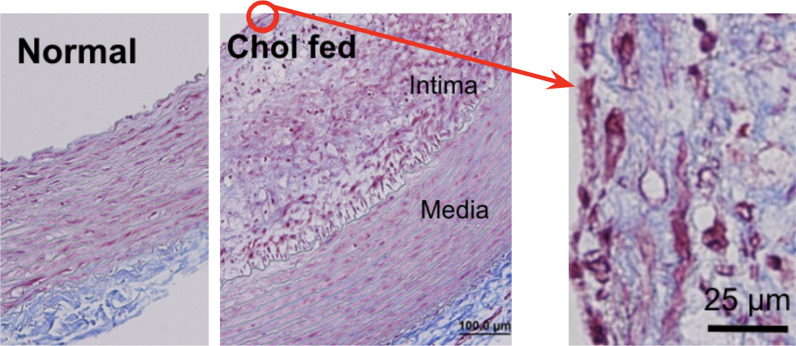
Supplemental histology for Fig. 2

**Fig. 12:**
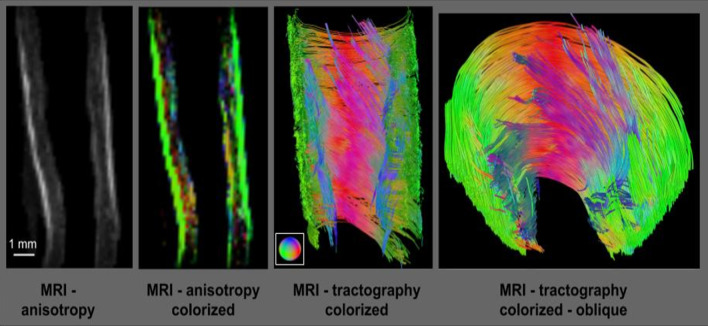
Anisotropic diffusion properties and meso-scale fiber orientation with tractography in long axis views obtained from GQI in the highly inflamed aorta of 1% cholesterol diet + injury rabbits. Long axis views obtained from GQI are shown. Tractography colors are encoded to fiber orientation as shown on the inset axis (oblique view). The scale bar size shown is 1 mm

To complement our methods and novel results for the vessel wall with GQI, we studied diffusion properties of the major atherogenic lipid in plaques (CE) by DWI combined with chemical resonance profiling under voxel-guided CMRS (Fig. 9). Without diffusion weighting (B = 0), all proton-rich regions of the vessel wall appear bright in CMR, whereas at high diffusion-weighting (high B-value), lipid-rich plaque areas remain bright, as can be seen in the overlay image of Fig. 9A. Diffusion signal intensity in the normal vessel wall areas decayed rapidly to 5% of the original signal at 4000 s/mm^2^, whereas in the plaque regions, diffusion signal intensity decayed to 76% of the original signal. The normal vessel wall area is dark in images at high B-value (Fig. 9A). Intensities measured at incremental degrees of diffusion weighting (500 s/mm^2^) reveal the differing diffusion properties of CE, the normal vessel wall, and water (Fig. 9B). CMRS was then used with voxels placed in the vessel wall regions of high and low signal intensity at high B-value to determine the chemical resonance profiles at those locations. The CMRS readings identified the presence of CE lipids in the DWI signal-intense regions and distinguished them from triglycerides (Fig. 9C).

## Discussion

We used DWI contrast and GQI methods of analysis for the purpose of quantifying multi-level vessel wall tissue architecture, subcellular details, orientation of vessel components, the presence of CE, and organizational properties in whole tissue. GQI analysis with tractography excels in characterizing meso-scale architecture, through the incorporation of intra-voxel and inter-voxel diffusion information, enabled by PDF generation [27, 31, 44] and tractography [25, 28, 29, 45]. Unique to this study, we showed that diffusion contrast arising from diffusion-restricting cell structures are confounded by contributions from slow diffusing CE lipids, while optimal design of DWI contrast differentiated the plaque molecular signals. Our results further differentiated lipid-rich regions from normal vessel wall tissues and cells. In addition, SMC constitute the primary cellular constituent of the vessel wall and are normally present in the media, where they are diffusion restrictive and anisotropic. During the process of atherosclerotic plaque progression, SMC migrate to the intima and can contribute to either progression or stabilization of the plaque, depending on the inflammatory status [5, 8, 9, 46, 47]. We observed SMC and ECM organization and co-alignment patterns in the thickened atherosclerotic intima that were well-differentiated from the media (Fig. 4).

Medial SMC were aligned perpendicular to the direction of blood flow, whereas the intimal SMC tended to align parallel along the length of the aorta. SMC alignment has previously been shown by the intensity gradient of cultured cells to be dependent on the magnitude and exposure time to shear stress [48], a possible explanation for the differences in SMC alignment with respect to vessel-wall location and depth. In a study of carotid atherosclerotic plaques by DTI, the authors hypothesized that longitudinally aligned SMC derives from the nearby regions, in contrast to SMC in deeper plaque locations from the tunica media with circumferential fiber orientations [49]. In our study, the SMC in each vessel wall region were shown to be co-aligned, resulting in meso-scale coherence across the respective aortic region (S1), although the intima had lower anisotropic diffusion properties compared to the media (Fig. 6). Thus, the degrees of SMC and ECM orientation and alignment were likely key steps in the process of inflammation, cell migration, and eventual wound healing. By comparison, short range anisotropy was observed in the lipid-rich thrombus (Fig. 8) but not meso-scale coherence as in the SMC and ECM rich vessel wall.

This study uniquely incorporates CE molecular and structural imaging using DWI contrast mechanisms. Previous applications of 2D DWI to atherosclerotic plaques have been optimized to locate CMR-visible CE or cells based on diffusion properties, but not both. CE comprised up to ~ 40% of plaque tissue by weight in our previous studies [50], which provides substantial proton density and contrast to the vessel wall. Although CE are “mobile,” they are present in viscous lipid droplets that are well differentiated from abundant, faster diffusing water. SMC membrane compartments are also diffusion restrictive, so these lipid and cellular components each contribute to the DWI signal. By further tuning the CMR B-value (diffusion time and gradient strength), it was possible to decouple the degree of proton-diffusion restriction contributed from CE lipids, in contrast to normal vessel wall cellular constituents or free water (Fig. 9). Using these methods as a template, it is feasible to identify lipid restrictive diffusion properties and cellular orientation in atherosclerotic vascular tissues.

While the entire experiment was complex with a sex difference in sample size (5 males, 3 females), and repetitions were constrained, the numbers of rabbits provided statistically significant data to support our interpretations. No difference was observed between male and female rabbits.

An important consideration for future studies is whether the methods utilized here will be translatable in vivo. A recent report applied slice-based DWI with apparent diffusion coefficient (ADC) maps for visualization of the aging thrombus in patients with deep vein thrombosis [51]. Relevant to our goals, DWI may enable identification of lipid-rich thrombus near the site of plaque rupture (e.g. white versus red thrombus) [52]. In addition, black-blood and motion-compensated DWI methods and sequences have previously enabled more accurate diffusion-based assessment of the vessel wall and lipid constituents. Since cardiac gated diffusion pulse sequences have been developed, it should be feasible to apply this approach in vivo for imaging of the human aorta. The question of maximum achievable geometric resolution will need to be determined experimentally, but the architectural analysis of plaques in the wall of the aorta should be achievable [53, 54]. From published methods, it is also possible to shorten the scan time for in vivo cardiac gated diffusion to a clinically feasible standard [54]. Moreover, in a study by Opriessnig et al., it was shown in single-slice measurements of healthy volunteers that it was possible to obtain 2D DTI scans of the aorta in vivo with 3 b-values and 18 gradient directions [55]. With improvements in gradient power, motion compensation methods, and pulse sequence design of clinical scanners, it may be possible to perform detailed 3D DTI or high-angular resolution GQI in the atherosclerotic vessel wall in vivo.

## Limitations

A limitation of our study is that 3D structures obtained from our CMR methods (and depicted by diffusion) are difficult to align with 2D morphology revealed by our histology because the 3D images necessarily cross dimensions as well as spatial scale. This is an area of ongoing research, but our image comparisons of fiber alignment portrayed serve the indicated purpose at this stage of our work.

## Conclusion

DTI CMR offers a potential method for the early diagnosis of progressive atherosclerotic disease. Because tissue (and plaque) attributes are difficult to ascertain by conventional imaging methods, disease monitoring is often based on indirect measurement methods. GQI showed new features of the luminal thrombus and characterization of the underlying atherosclerotic vessel wall. Thus, early detection of micro- and meso-scale level vascular destabilization using GQI could increase the accuracy of diagnosis and assessment of treatment outcomes in individuals with atherosclerosis. By using diffusion-based CMR contrast mechanisms with variation of magnetic field gradient orientation and strength, diffusion measurements can be made for the analysis of vessel wall anisotropic and diffusion restrictive properties. The ability to visualize vessel wall diffusion properties gives insights into linkages between SMC and ECM vessel wall architecture, the presence of CE lipids in plaques, and thrombus properties, thus providing new understanding about the progression from lipid deposition to thrombosis that underlies the clinical complication of atherosclerosis.

## Data Availability

The data that support the findings of this study are available from the corresponding author upon request.

## Appendix

### Generalized Q-space imaging (GQI)-derived quantitative diffusion metrics

Quantification (Table 1) was carried out to determine the degree of anisotropy and diffusion restrictive properties with respect to depth in the atherosclerotic vessel wall. These results are shown with individual readings from GQI metrics and with detailed statistical results. The enlarged intima had lower diffusion anisotropic properties than the media (shown by generalized fractional anisotropy (GFA)), while other GQI metrics showed higher restrictive (RDI), isotropic (ISO), and non-restrictive (nRDI) diffusion properties of the intima. Anisotropy with diffusion tensor imaging (DTI) fractional anisotropy (DTI-FA) was calculated for comparison, showing similar trends of higher anisotropy in the media, but with higher variability between readings compared to GFA (Figs. 10, 11 and 12).

## Abbreviations

ADC: Apparent diffusion coefficient
CE: Cholesteryl esters
CMR: Cardiovascular magnetic resonance
CMRS: Cardiovascular magnetic resonance spectroscopy
DTI: Diffusion tensor imaging
DTI-FA: Diffusion tensor imaging fractional anisotropy
DWI: Diffusion-weighted imaging
ECM: Extracellular matrix
EPI: Echo planar imaging
FA: Fractional anisotropy
GFA: Generalized fractional anisotropy
GQI: Generalized Q-space imaging
ISO: Isotropic diffusion component
nRDI: Non-restricted diffusion index
ODF: Orientation distribution function
ORO: Oil Red O
PDF: Probability distribution function
PLM: Polarized light microscopy
RDI: Restricted diffusion index
ROI: Regions of interest
SMC: Smooth muscle cells
